# Southern Surgical and Gynecological Association

**Published:** 1893-01

**Authors:** 


### SOUTHERN SURGICAL AND GYNECOLOGICAL ASSOCIATION.

*Fifth Annual Meeting, held in Louisville, Kentucky, November 15, 16, and 17, 1892.*

(Continued.)

DR. HENRY O. MARCY, of Boston, followed with a paper on "The Cure of Inguinal Hernia in the Male," in which he said until recently the cure of inguinal hernia in the male had been considered at the best accidental, and when apparently affected, generally doubtful, and the hernia liable to return. The great majority of surgeons looked upon an attempt to cure as ill-advised, and believed operative measures should not be undertaken except in cases of strangulation. Dr. Marcy thought there was abundant reason for such conclusion, when judged from the earlier history of surgical procedures as attempted for cure. The essential surgical considerations for the cure of hernia were as follows: First—Strict aseptic conditions. These pertain alike to all modern surgical procedures. Second—A free dissection. This is necessarily in order to lay bare the internal ring, to permit of the enucleation of the peritoneal sac, and the separation and elevation of the cord out of the wound. The external epigastric artery often courses in the line of the incision. It is not seldom that the size of this vessel is such that the operator fears he has wounded the larger vessel. Third—The disposition of the sac. The separation of the sac to its very base before removal is to be recommended as the rule. There are times, however, when it is not easy to free the peritoneal pouch, owing to the adhesion to the surrounding tissues, and in large old irreducible hernia a more or less intimate fusion of the contents to the inner wall of the sac. It is generally better to open the sac before ligating or sawing through its neck, since by so doing the condition at the internal ring is assured and by such knowledge the operator is often profited, even if the sac is completely empty, not seldom the omentum is adherent at the internal ring and even a constricted loop of intestine may escape observation when it is attempted to return the sac unopened.

DR. MARCY closed his paper by saying that between three and four millions of the people living in the United States were subject to hernia; and, if the demonstration is complete that the risk of life is less than one per cent. from the operative procedures instituted for cure, and that scarcely more than ten per cent. are subject to relapses, and these almost invariably in a state improved by the operation, the plea is a very strong one, to consider favorably the advisability of operation in a very large majority of all the sufferers from hernia.

DR. W. H. WATHEN, of Louisville, read a paper entitled "The Treatment of Umbilical and Ventral Hernia." He said the importance of studying carefully the best methods of treating hernia is now especially emphasized because of the increased frequency of the disease following laparotomy, and especially because the modern methods of surgery make the operation far less dangerous than it was formerly. The operation for radical cure of hernia in the practice of the best surgeons, except in extreme cases, is practically devoid of danger, and the result may be made permanent. Modern antiseptic and aseptic precautions have practically excluded the danger which formerly arose from infective peritonitis. The author said there are many cases of ventral hernia that could have been prevented had the proper treatment been carried out in the closure of the abdominal wound. In order that there may be no hernia following laparotomy it is necessary to get perfect union by adhesion of all the layers of tissue forming the abdominal wall—the peritoneum, muscles, the deep and superficial fascia, and the skin. But especially must we get union of the layers of fascia, for unless this be done the other layers will gradually separate and hernia will follow. This cannot be done unless we succeed in bringing the cut edges of the fascia in even and perfect apposition long enough for strong union to occur.

### SECOND DAY—MORNING SESSION.

DR. W. O. ROBERTS, of Louisville, read a paper on "The Treatment of Ununited Fractures by Resection." He said the treatment of ununited fractures by resection was more than a hundred years old, White of Manchester having done the first operation in 1760. In consequence of the great mortality attending the operation it was abandoned until revived by Sir Benjamin Brodie. In 1805 Horeau, after having divided the fragments, obliquely fastened them together by tying a metallic wire around them. Rogers, of New York, in 1838, passed the wire through holes drilled in the wall of the fragment and then twisted it. Since then other surgeons have used sutures of various materials in the same way. Some of them leaving the sutures in permanently, while others removed them after union of the fragments had occured. Some instead of drilling the bone passed the sutures simply through the periosteum. Screws, nails, ivory pegs and clamps have been used for the same purpose. In the long bones when coaptation of the fragments can be secured, Dr. Roberts feels satisfied that resection and a fixed dressing will be followed by just as good results as when sutures or other contrivances for fastening the ends of the fragments together are used.

"Symptoms of Fracture, their Importance and Significance."

Dr. W. C. Dugan, of Louisville, read a paper on this subject. (Abstract of same not received.)

DR. BEDFORD BROWN, of Alexandria, Va., related the case of a boy who sustained a compound comminuted fracture of the skull in 1860, yet he was perfectly conscious and had no symptoms of compression of the brain. The spiculæ of bone were removed, and recovery followed.

DR. J. H. LETCHER, of Henderson, Ky., advised against the too hasty resort to the use of the trephine and chisel in injuries of the skull.

DR. C. KOLLOCK, of Cheraw, S. C., had trephined in two cases with successful results.

DR. J. H. MCINTYRE, of St. Louis, reported a case of traumatic insanity in a railroad employe in which he trephined with success. The fracture was an extensive one and occurred in the upper Rolandic region. He reported several other interesting cases.

DR. WILLIAM WARREN POTTER, of Buffalo, called attention to fracture of the internal table of the skull without fracture of the external; hence the great liability to error in diagnosis.

DR. HOWARD A. KELLY, of Baltimore, related the case of a man who fell and was brought to the Presbyterian Hospital in a comatose state. Careful examination revealed the fact that the man had diabetic cataract with fracture at the base of the skull.

DR. WILLIAM T. BRIGGS, of Nashville, had trephined in fifty cases of epilepsy. Four-fifths of the cases operated on were relieved temporarily but not permanently.

DR. T. W. REAMY, of Cincinnati, mentioned the case of a man who had fallen from the second story of a court house, sustaining a fracture of the skull, but had never had epilepsy or any bad symptom following the injury. He thought this case would be some comfort to country practitioners who did not enlarge the scalp wound in all cases.

The papers were further discussed by Drs. Vance, Lydston, Nicolson, Greenly and Baxter, all of them favoring radical measures in the treatment of injuries of the skull.

DR. L. S. MCMURTY, of Louisville, read a paper enlitled "Ovarian Cystoma with Twisted Pedicle and Peritonitis; Ovariotomy in Second Week of Typhoid Fever—Recovery." (No abstract received of this paper.)

DR. H. HORACE GRANT, of Louisville, contributed a paper on "Intestinal Anastomosis by a New Device." For more than a year the speaker has been endeavoring to perfect some instrument to simplify suture, but it has been so difficult to get just what he wanted that time has not been allowed since the completion of the instrument to test it fully. It is to be used only after resections. The two blades of the clamp are oval scissors, one-fourth of an inch in transverse and two and a half inches in longitudinal diameter. The arms of the clamp are made long enough to allow of introduction full five inches. After the gut is exposed, a strand of iodoform gauze is passed through the mesentery and constricts the intestines fully six inches from each point of intended resection. The mesentery is tied off over the portion to be resected with fine silk, in two inch loops, cut close and dropped in the usual way. When the resected portion is removed the gut ends may be washed out if desired. While the two ends of the divided intestine are held parallel, one blade is entered in each, allowing at least one and one-half inches of gut beyond the proposed anastomotic opening to permit of invagination of the ends. The clamp is tightened and the two surfaces thus firmly held are rapidly stitched together by a continuous overhand Lembert suture of fine silk. Two rows of parallel sutures as suggested by Abbe may be used if desired, though it has seemed that one is enough according to the author's experiments. The work can be done far more rapidly and accurately than without the clamp. When the suturing is finished the clamp is tightened if need be, and a long bladed dressing forceps passed in the bowel and the oval plug removed or pushed in. The scissors action of the blades, together with the ten or fifteen minutes pressure prevents any hemorrhage. The clamp is now withdrawn and the ends invaginated in the usual way.

DR. A. V. L. BROKAW, of St. Louis, thought the instrument exhibited by Dr. Grant, a good one, and said that anything which materially assists the surgeon in making intestinal anastomotic operations rapidly was of great value, that time was a most important element. The use of rings, plates and mats in the past were bad. He believes that we can suture far more rapidly with Dr. Grant's instrument than any other device he has thus far seen.

Dr. W. E. B. Davis, of Birmingham, Ala., believed a large number of operators had abolished mechanical devices in doing intestinal anastomosis. His brother, Dr. John D. S. Davis, had devised a rubber plate and mat, but now prefers not to use the plate. In the case of restriction of the bowel, he thought the device of Dr. Grant was an ingenious one, inasmuch as it would facilitate the work of the surgeon and enable him to do an operation very quickly. He had conducted a series of experiments in an effort to do away with mechanical devices, by which surgeons might use the end to end operation by splitting up the bowel. While the operation was successful in some cases, the strain on the circulation was too great, and he now condemns the operation.

Dr. G. Frank Lydston, of Chicago, directed attention to Dr. J. B. Murphy's anastomosis button, a recent device by which he says cholecysto-enterostomies can be done in from eight to twelve minutes.

SECOND DAY—AFTERNOON SESSION.

The Association was called to order at 2:30 p. m., with the First Vice-President, Dr. C. Kollock, in the chair.

The President delivered his Annual Address. He took for his subject "Compatibility of Conservative and Aggressive Surgery."

Dr. Ap. Morgan Vance, of Louisville, read a paper entitled "A Plea for More Rapid Surgical Work," in which he said a great number of surgeons pay little attention to the time consumed in an operation, or to the nicety of manipulation and dextrous use of instruments that our forefathers prided themselves upon. He had seen on numerous occasions the work of our most distinguished surgeons, and seen deaths occur from prolonged anæsthesia and too much time consumed in operation which would not have taken place if much unnecessary time had not been wasted. The habit of starting the anæsthetic before all preparations were completed was very reprehensible.

The paper was discussed by Drs. Arch. Dixon, Rufus B.

Hall, Geo. A. Baxter and Edwin Ricketts, all endorsing the position taken by the essayist.

DR. CHARLES A. L. REED, of Cincinnati, contributed a paper entitled "Surgery of the Ureters with Report of Cases."

He said that surgery of the ureter is one of the developmental subjects of abdominal surgery. These out-of-the-way conduits exercising functions that are vital in character were liable to diseased conditions which baffle the resources of the diagnostician and tax the ingenuity of the operator. For purposes of diagnosis the physical means at our disposal may be briefly summarized as follows: (1) Exploration of the lower end of the ureter by digital examination, (a) through the vagina, (b) the rectum, and (c) the bladder; (2) exploration of the lower end of the ureters by the sound passed through the urethra and bladder into the ureters; (3) exploration of the central portion of the ureters by abdominal lumbar palpation —an expedient of practical value only in cases of extreme ureteral distension occuring in very thin subjects; (4) exploration of the upper end of the ureters by exploratory nephrotomy. Each of these several expedients might be amplified, but it would be uncalled for in the presence of such distinguished members. Dr. Reed said that since catheterization of the ureters has been popularized in this country chiefly through Kelly, and since the technique of the procedure has become understood by those who have studied it, the diagnosis of disease within and surrounding these tubes if vastly more common. The digital exploration of that portion of the ureters lying within easy reach from the vagina or rectum is readily practiced by those who have carefully studied the anatomy of the parts. Digital exploration through the urethra and bladder is an easy expedient so far as the surgeon is concerned and often leads to the elucidation of important pathological facts, but the speaker is forced to believe that it is not without danger to the patient. He has been forced into this belief by one case of incontinence lasting for nearly a year and by two cases of weakened power of retention, one of which is now of quite two years standing. Dr. Reed said that abdominal section for diagnosis of ureteral conditions, notably in cases of suspected calculus, is entirely justifiable. He then reported a case of periureteritis; stricture; kolpo-cysto-ureterotomy, with recovery. The second case was one of cicatricial stricture of an excised ureter; hydro-nephrectomy; remaining urethral disease.

DR. WILLIAM WARREN POTTER, of Buffalo, read a paper entitled "Specialism in Medicine Particularly as Related to Surgery and Gynecology." He summarized his argument thus:

1. There is essential need for specialists. Divisions of labor in every field are demanded, and nowhere more than in medicine.

2. Specialists being a necessity, they must equip themselves by years of study, and devote themselves to a still greater number of years of general practice before they are justified in offering themselves as specialists.

3. They must conduct themselves in such a way as to merit the respect of the general practitioner, and to invite his co-operation in their work.

4. The unwritten ethics of specialism demand that there shall be reciprocal relationship maintained not only among specialists themselves, but also between specialists and general practitioners.

5. The opportunities for perfection in special lines of medical study are so great, and medical literature in both journalistic and text-book form is so rich, that an awful responsibility is entailed.

6. The schools ought to discourage any and all students who give promise of entering upon the practice of a specialty as soon as the college doors are passed, and before the swaddling clothes of the professional tyro are shipped.

DR. R. M. CUNNINGHAM, of Birmingham, Ala., followed with a paper entitled "The General Practitioner as a Gynecologist." He said the general practitioner should not undertake work that can be better and more safely done by the specialist, provided one is obtainable. He should be willing to do and attempt the most radical and dangerous operations when necessary to save life, provided a specialist or one better prepared to do the work cannot be obtained. In cases not necessarily dangerous, or in which life does not become more or less a burden, but in which a cure can be effected only by a radical procedure, but which may be materially benefited, or symptomatically relieved by milder methods, he should adopt the latter and not the former. In many cases the field is clearly his own, belongs to him, and he should be prepared and competent to treat them with safety and success.

DR. W. F. WESTMORELAND, of Atlanta, read a paper on "Specialism in Medicine."

He said there were two kinds of specialists, the one with his preconceived ideas, which become warped, who always suffers from astigmatism, etc., who is a graduate of his kind. The other kind was the man who has worked his way by his generally acknowledged ability in any particular line.

DR. HOWARD A. KELLY, of Baltimore, read a paper entitled "A Preliminary Report on the Morphology of Ovarian and Myomatous Tumors.

The speaker said the form of abdomen characteristic of large ovarian cysts is a globular or ovoid distention of a part or the whole of the abdominal wall, pushing out the infra-umbilical portion much more than the supra-umbilical, at least so long as the tumor occupies the lower half or two-thirds of the abdomen. This enlargement is uniform in parovarian cysts and polycystic tumors, exhibiting but few bosses, due to the fact that the latter are composed of one or two large cysts associated with a mass of smaller ones, and the large cyst is best accommodated in the median line in the distended concave anterior abdominal wall, while the smaller ones at the side or back consequently do not show. Prominent exceptions to the general rule just enunciated that pelvic tumors distend most markedly the inferior abdominal zone are the notable stretching of the upper abdomen in very fat women with large ovarian tumors, and the like distention in rachitic dwarfs in advanced pregnancy. Nodular myomata, on the other hand, stand out in marked contrast to the smooth outlines of cystic tumors in giving to the lower abdomen a lumpy bossed appearance, thus exhibiting through muscles and skin a softened exaggerations of their irregular outlines. This peculiarity still remains prominent although softened, after these tumors have undegone fibrocystic degeneration.

